# The Effect of Polysaccharides and Saponins from *Polygonatum kingianum* on Cognitive Dysfunction in an AlCl_3_-Induced Alzheimer’s Disease Zebrafish Model

**DOI:** 10.3390/foods15101785

**Published:** 2026-05-18

**Authors:** Jiawei Yu, Yike Jiang, Yang Li, Bei Fan, Cong Lu, Meng Tang, Wenbiao Luo, Yiqing Yang, Fengzhong Wang, Qiong Wang

**Affiliations:** 1 Institute of Food Science and Technology, Chinese Academy of Agricultural Sciences, Beijing 100193, China; ycloverjswmart@163.com (J.Y.); jiangyikezz@163.com (Y.J.); engsut1@gmail.com (Y.L.); fanbei517@163.com (B.F.); lucong198912@126.com (C.L.); 2 National Nanfan Research Institute (Sanya), Chinese Academy of Agricultural Sciences, Sanya 572024, China; 3 Research Institute of Polygonatum Kingianum Industry, Huaihua 419200, China; tangmeng2026@163.com (M.T.); lwb2926@126.com (W.L.); xhlyjbgs2021@163.com (Y.Y.)

**Keywords:** Alzheimer’s disease, *Polygonatum kingianum*, acetylcholinesterase, amyloid-beta aggregation, neuroprotection

## Abstract

*Polygonatum kingianum* (PK), a plant with established medicinal and nutritional applications, has shown potential neuroprotective activity in Alzheimer’s disease (AD). Nevertheless, the effects of its major bioactive fractions remain unclear. This study examined the neuroprotective effects of PK polysaccharides (PKPs) and saponins (PKSs) using an AlCl_3_-induced zebrafish model. Chemical analyses revealed that PKP was dominated by a low-molecular-weight fraction (1890 Da, 83.8%), whereas LC-MS analysis detected 13 tentatively identified steroidal saponins within PKS, including diosgenin. Furthermore, behavioral assessments demonstrated that both PKP and PKS improved locomotor and cognitive functions. PKP exhibited a stronger effect on the cholinergic system; its acetylcholinesterase (AChE) inhibitory activity at 60 μg/mL was comparable to that of donepezil under the experimental conditions. Histopathological analysis indicated that PKP showed a stronger effect in reducing neuronal apoptosis, resulting in a 68% reduction in the number of apoptotic cells. Conversely, PKS displayed a greater effect on amyloid pathology, reducing amyloid-beta (Aβ) aggregation by 62%. These findings suggest that PKP and PKS showed different neuroprotective profiles in the zebrafish model. Specifically, PKP was more closely associated with cholinergic regulation and neuronal survival, whereas PKS showed a stronger effect on Aβ aggregation. This study provides experimental support for the potential use of PK-derived fractions as food-derived bioactive components for alleviating AD-related pathological changes.

## 1. Introduction

Alzheimer’s disease (AD) is the main cause of dementia, accounting for about 60% to 70% of all cases worldwide. This neurodegenerative condition is characterized by a progressive decline in cognitive and behavioral functions, affecting memory, decision-making, and overall daily activities [1]. AD is characterized by the widespread compromise and atrophy of neuronal networks essential for cognitive processing, with neuronal loss resulting in a gradual erosion of memory and behavioral stability [2]. While medications like aducanumab and lecanemab can help manage the symptoms of Alzheimer’s disease (AD), a complete cure has yet to be found. The broader use of these therapies is hindered by their high cost and the potential for serious side effects [3]. As a result, there is an increasing demand for natural alternatives that are both safe and cost-effective for AD treatment.

Interest in edible plants containing bioactive macromolecules has been rising, driven by the increasing demand for natural alternatives. Polygonatum, a genus within the Asparagaceae family, is prevalent across temperate zones in the Northern Hemisphere, encompassing China, Japan, South Korea, India, Russia, Europe, and North America [4,5]. The diverse bioactive constituents present in Polygonatum are associated with a wide array of health advantages, including sleep enhancement [6], anti-aging [7], anti-fatigue [8], antibacterial [9], immunomodulatory [10], lowering blood sugar [11], anti-cancer [12], hepatoprotective [13] and anti-atherosclerotic effects [14]. Furthermore, recent findings have underscored the neuroprotective capabilities of Polygonatum’s bioactive compounds, which exert their effects through multiple pharmacological mechanisms. Recent investigations have demonstrated that Polygonatum polysaccharides, particularly those derived from traditional steaming techniques, can substantially mitigate memory deficits in D-galactose-induced aging models. These observed benefits are associated with increased antioxidant capacity, diminished neuroinflammation, and improved synaptic functionality, possibly implicating the Nrf2/HO-1 signaling pathway [15]. Moreover, Polygonatum saponins have been shown to counteract the competitive inhibition of muscarinic (M) receptors by scopolamine, thus facilitating cholinergic neurotransmission and enhancing cognitive performance in experimental settings [16]. *Polygonatum kingianum*, in particular, has attracted considerable interest within this genus, owing to its high polysaccharide and saponin concentrations, coupled with its historical application as both a therapeutic and consumable plant [17,18]. As detailed in a recent comprehensive review, *P. kingianum* is characterized by its specific steroidal saponins and fructose-rich polysaccharides, which undergo significant structural and functional changes during traditional processing [19]. Based on these previous findings, the current study focuses on *Polygonatum kingianum* Coll. et Hemsl., which is included in the Chinese Pharmacopoeia as one of the original sources of Polygonatum medicinal materials [20].

Zebrafish, owing to their genetic proximity to humans, their optical clarity during early developmental stages, and their amenability to high-throughput screening, have emerged as a significant model organism in neuropharmacological investigations [21]. Specifically, the neurotoxicity induced in zebrafish by AlCl_3_ has been extensively employed to simulate various Alzheimer’s disease (AD)-like characteristics, encompassing cholinergic dysregulation, the accumulation of amyloid-beta (Aβ), and demonstrable cognitive deficits [22]. Therefore, the aim of the present study is to isolate and characterize PKP and PKS from processed *P. kingianum* and to comparatively evaluate their neuroprotective effects using an AlCl_3_-induced zebrafish model. By assessing behavioral changes and biochemical markers, we seek to elucidate the distinct pharmacological tendencies and potential synergistic value of these two major fractions.

## 2. Materials and Methods

### 2.1. Materials

Fresh rhizomatous tubers of *Polygonatum kingianum* (PK) were provided by Huairen Group Bozhikang from Hecheng District, Huaihua City, Hunan Province, in December 2024. It was scientifically identified as *Polygonatum kingianum* Coll. et Hemsl. by Researcher Yibo Luo from the Institute of Botany, Chinese Academy of Sciences. A voucher specimen (No. PK-20241201) has been deposited in the Herbarium of Chinese Academy of Agricultural Sciences.

### 2.2. Preparation and Characterization of PKP

#### 2.2.1. Extraction and Purification of PKP

PK was sliced into 5 mm thick pieces and processed by steaming for 3 h, followed by four cycles of alternating steaming and drying at 55 °C for 3 h. The material was then freeze-dried, ground into powder, and passed through a 60-mesh screen. In total, 90 g of the powder was extracted with distilled water (1:20, *w*/*v*) in a water bath at 80 °C for 2 h. After two additional extractions, the combined extracts were concentrated to 30% of the original volume using a rotary evaporator(Greatwall, Zhengzhou, China) at 55 °C. Anhydrous ethanol was added to achieve a final concentration of 80% (*v*/*v*), and the mixture was left at 4 °C for 12 h. The precipitate was collected by suction filtration and washed three times with anhydrous ethanol and acetone. Proteins were removed using the Sevag method [23] (chloroform:n-butanol = 4:1 *v*/*v*; reagent:polysaccharide solution = 1:3 *v*/*v*), and pigments were adsorbed with activated carbon [24]. The solution was dialyzed against distilled water for 3 days using a membrane with a molecular weight cut-off (MWCO) of 1000 Da, with the water changed every 12 h. The solution was then freeze-dried. The crude polysaccharide was then further purified by loading onto an AB-8 macroporous resin column and eluting with 5 column volumes (CV) of distilled water. Finally, the water eluate was concentrated at 55 °C and vacuum-dried to yield the purified *Polygonatum kingianum* polysaccharide (PKP). The total carbohydrate content of the obtained PKP was 65.0%, as determined by the phenol-sulfuric acid method.

#### 2.2.2. Determination of the Molecular Weight of PKP

The molecular weight distribution of PKP was analyzed by high-performance gel permeation chromatography (HPGPC) [25]. The analysis was performed on a system with an RID-20A refractive index detector (Shimadzu, Kyoto, Japan) and a BRT105-103-101 gel column (8 mm × 300 mm; Borui Saccharide, Yangzhou, China). To prepare the test solution, 5.00 mg of PKP was dissolved in 1 mL of the mobile phase (5 mg/mL), vortexed, and centrifuged at 12,000× *g* for 10 min at 4 °C. The supernatant was then filtered through a 0.22 μm hydrophilic membrane before analysis. A 0.5 M NaCl solution was used as the mobile phase at a flow rate of 0.7 mL/min and a column temperature of 40 °C, with an injection volume of 100 μL. A series of Dextran standards (5 mg/mL) were analyzed under the same conditions to establish mathematical models by plotting the logarithm of molecular weights (log Mp, log Mw, and log Mn) against retention time. The key molecular weight parameters (Mp, Mw, and Mn) of PKP were subsequently calculated based on these standard curve equations.

#### 2.2.3. Determination of Monosaccharide Composition

The monosaccharide composition of PKP was determined by ion chromatography after acid hydrolysis [26]. Specifically, 2 mg of PKP was hydrolyzed with 2 mL of 3 M trifluoroacetic acid (TFA) at 80 °C for 2 h. After hydrolysis, the solution was dried under a nitrogen stream, re-dissolved in 5 mL of ultrapure water, and mixed thoroughly. A 50 μL aliquot was diluted to 1 mL and centrifuged at 12,000 rpm for 5 min. An IC analysis was performed on a Dionex Carbopac PA10 column (250 mm × 4 mm; Thermo Fisher Scientific, Waltham, MA, USA) at 30 °C. The mobile phase consisted of A (ultrapure water), B (15 mM NaOH and 100 mM NaOAc), and C (15 mM NaOH) at a flow rate of 0.3 mL/min with an injection volume of 25 μL. An electrochemical detector was used for signal acquisition. Monosaccharide standards included fucose (Fuc), galactosamine (GalN), rhamnose (Rha), arabinose (Ara), glucosamine (GlcN), galactose (Gal), glucose (Glc), N-acetylglucosamine (GlcNAc), xylose (Xyl), mannose (Man), fructose (Fru), ribose (Rib), galacturonic acid (GalA), guluronic acid (GulA), glucuronic acid (GlcA), and mannuronic acid (ManA).

#### 2.2.4. Fourier Transform Infrared (FTIR) Spectrum Analysis

The organic functional groups of PKP were identified using an FT-IR650 spectrometer (Gangdong, Tianjin, China). Samples were prepared by mixing 2 mg of PKP with 200 mg of KBr. The mixture was pressed into a translucent pellet for analysis. A pure KBr pellet served as the blank control. The spectra were recorded over a scan range of 4000–400 cm^−1^ with a resolution of 4 cm^−1^ and 32 scans per spectrum.

### 2.3. Preparation and Characterization of PKS

Saponins from PK were extracted using an ultrasonic-assisted extraction method to obtain bioactive constituents. In total, 90 g of PK powder was extracted with 80% ethanol at a solid-to-liquid ratio of 1:20 (*w*/*v*). The mixture was allowed to stand for 30 min before undergoing ultrasonic extraction at 480 W and 70 °C for 1 h. After vacuum filtration, the extraction procedure was repeated. The combined filtrates were concentrated under reduced pressure to remove ethanol, and lipid-soluble impurities were removed by petroleum ether. The aqueous phase was then extracted three times with water-saturated n-butanol using countercurrent extraction. The organic layers were combined, concentrated, and dried. The resulting residue was dissolved in 80% aqueous methanol, filtered, and purified using AB-8 macroporous resin. After elution with 95% aqueous ethanol, followed by concentration and drying, the *Polygonatum kingianum* saponins (PKSs) were obtained. The composition of PKS was analyzed using a Waters ACQUITY UPLC HSS T3 column (2.1 mm × 100 mm, 1.8 µm; Waters Corporation, Milford, MA, USA). The mobile phase consisted of A (acetonitrile) and B (0.1% formic acid) at a flow rate of 0.3 mL/min. The MS was operated in ESI mode with a scan range of 50–1700 *m*/*z* and collision energies of 20, 40, and 60 eV.

The quantification of Dioscin in PKS was performed using LC–MS. A standard curve was established using an authentic Dioscin standard (purity ≥ 98%) at concentrations ranging from 0.5 to 50 µg/mL. The regression equation was y = 13780.57x + 431.47, with a correlation coefficient (R^2^) of 0.9995, indicating excellent linearity. The content of Dioscin in the PKS fraction was then calculated based on the peak area.

### 2.4. Animal Experiments

#### 2.4.1. Animals and Embryos

Wild-type AB strain zebrafish were obtained from the National Zebrafish Resource Center (Wuhan, China). These fish were housed within a recirculating water system, where environmental parameters were meticulously controlled: temperature was maintained at (28.0 ± 0.5) °C, conductivity fluctuated between 400 and 500 μS/cm, and pH levels were kept between 7.0 and 7.6. A light cycle of 14 h of illumination followed by 10 h of darkness, commencing at 07:00, was implemented. All experimental protocols were conducted in accordance with the ethical standards outlined by the National Regulations for the Administration of Laboratory Animals.

Healthy embryos, 3 h post-fertilization (hpf), were randomly distributed into 6-well plates (30 embryos per well) and incubated at 28 °C. The medium was replaced daily, and debris was removed. To induce Alzheimer’s disease (AD) in the larvae, zebrafish at three days post-fertilization (dpf) were exposed to 150 μM acidic AlCl_3_ until they reached six dpf.

**Figure 1:**
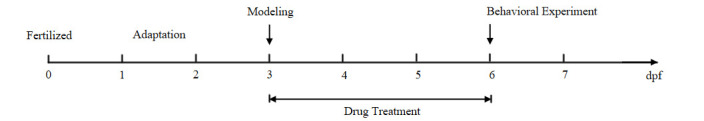
Experimental flowchart.

The experimental design included seven different groups: a control group (CON), a model group (MOD) exposed to 150 μM AlCl_3_, a positive control group (DPZ) treated with 5 μM Donepezil, and two sets of groups treated with PKP (60 and 120 μg/mL) and PKS (20 and 60 μg/mL), respectively. Throughout the treatment duration, spanning from 3 to 6 days post-fertilization (dpf), both the medium and the administered drugs underwent daily complete replacement.The detailed timeline of the in vivo zebrafish experimental procedure is illustrated in Figure 1.

#### 2.4.2. Light–Dark Alternation Behavioral Test

After 3 days of treatment, 6 dpf larvae were tested using the Noldus EthoVision XT system (Noldus Information Technology, Wageningen, The Netherlands). The larvae were transferred to 48-well plates (one fish per well) and allowed to acclimate under light conditions for 10 min. The formal behavioral test consisted of two 10 min dark/10 min light cycles, giving a total test duration of 40 min. Locomotor activity was continuously recorded throughout the acclimation and test periods (total recording time: 50 min). Total distance and average speed during the formal test period were calculated to evaluate the effects of PKP and PKS on AD-like behavioral deficits.

### 2.5. AChE Activity Assay

Acetylcholinesterase (AChE) activity was measured to assess cholinergic function using a commercial assay kit (Nanjing Jiancheng Bioengineering Institute, Nanjing, China) based on the Ellman method. Thirty 6 dpf larvae were collected per group into 1.5 mL tubes, with three biological replicates per group. The samples were homogenized on ice at a ratio of 1:10 (*w*/*v*) and centrifuged at 8000× *g* for 10 min at 4 °C. The supernatant was collected for AChE determination using a commercial assay kit, and absorbance was measured at 412 nm using a microplate reader(Shanghai Flash Spectrum Biotechnology, Shanghai, China). The total protein concentration of the supernatant was determined using a BCA protein assay kit (Beyotime, Shanghai, China) for normalization. AChE activity was normalized to protein concentration and expressed as U/g protein.

### 2.6. Thioflavin S Staining

After treatment, 6 dpf larvae were anesthetized with 0.03% tricaine and collected (n ≥ 6 per group). The samples were fixed in 4% paraformaldehyde (PFA) overnight (12–16 h) at 4 °C and then washed three times with PBS. After dehydration with graded ethanol (25% to 50%), the samples were incubated with 0.3% Thioflavin S for 60 min in the dark. The samples were then differentiated with 80% ethanol every 5 min until the background signal decreased. After rehydration in PBS, brain images were captured using a Nikon A1R confocal laser scanning microscope (Nikon, Tokyo, Japan). To ensure accuracy, Thioflavin S fluorescence was excited at 488 nm and detected at 525 nm (green), while DAPI counterstaining was used to locate nuclei (Ex 405 nm/Em 461 nm). All imaging parameters, including laser intensity and gain, were kept constant across groups. Thioflavin S-positive fluorescent deposits in the brain were quantified to evaluate Aβ aggregation.

### 2.7. TUNEL Staining

Brain cell apoptosis was detected using the TUNEL assay. After fixation, zebrafish larvae were embedded in paraffin and sectioned at a thickness of 5 μm. DNase I was used as a positive control. The sections were equilibrated in buffer for 10–30 min at room temperature and then incubated with 50 μL TdT incubation buffer containing TdT enzyme and FITC-labeled dUTP at 37 °C for 60 min in the dark. After washing with PBS, nuclei were counterstained with 2 μg/mL DAPI for 5 min. Sections were mounted with anti-fluorescence quenching mounting medium and imaged using a fluorescence microscope. Apoptotic cells (FITC-labeled, green fluorescence at 520 ± 20 nm) and nuclei (DAPI-labeled, blue fluorescence) were merged to count the number of apoptotic cells in the zebrafish brain.

### 2.8. Statistical Analysis

Statistical analyses were performed using Origin version 2021, GraphPad Prism version 9.0, and IBM SPSS version 26.0. Behavioral data are expressed as mean ± standard error of the mean (SEM). Other quantitative data are presented as mean ± standard deviation (SD). One-way analysis of variance (ANOVA) was used to assess differences among groups, followed by Dunnett’s multiple comparison test. A *p*-value of <0.05 was considered statistically significant, and *p* < 0.01 was considered highly significant.

## 3. Results

### 3.1. Characterization of PKP

**Figure 2:**
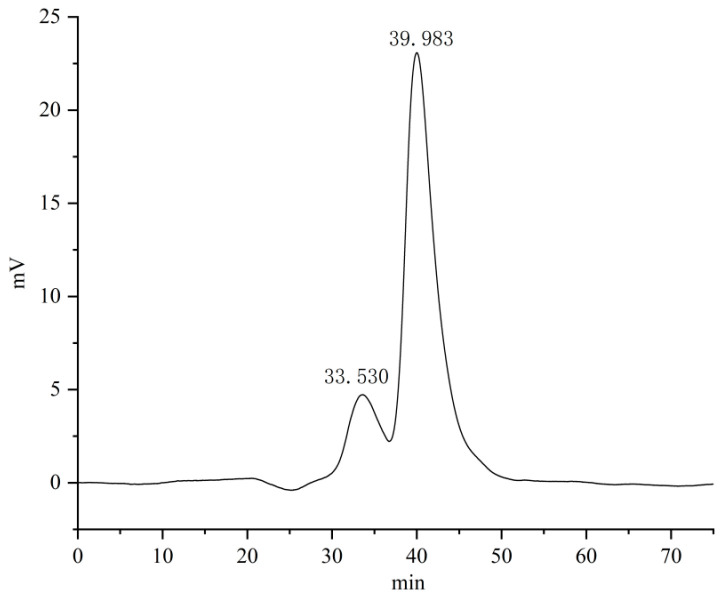
Molecular weight distribution of PKP determined by HPGPC.

The molecular weight distribution of PKP was determined using high-performance gel permeation chromatography (HPGPC) (Figure 2). The chromatogram exhibited a bimodal distribution. The principal peak, designated Peak 2, eluted at 39.98 min, constituting 83.82% of the aggregate peak area and possessing a molecular weight (Mp) of 1890 Da. Conversely, a secondary peak, Peak 1, eluting at 33.53 min and accounting for 16.18% of the total area, corresponded to a substantially greater molecular weight of 41,321 Da. As detailed in Table 1, the polydispersity indices (Mw/Mn) for the two fractions were 1.03 and 1.04, respectively. Additionally, a signal observed at 42.8 min was identified as a system peak.

**Figure 3:**
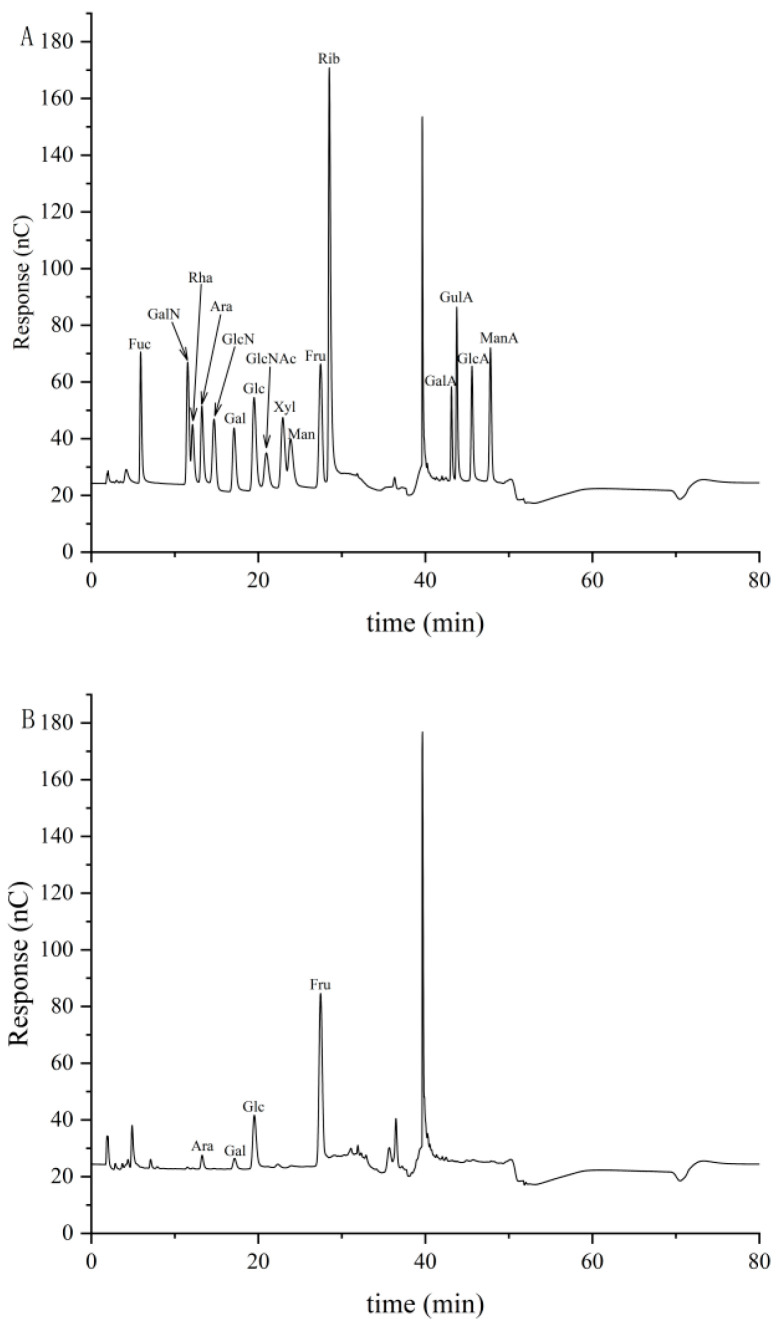
HPAEC-PAD chromatograms of monosaccharide standards and PKP. (**A**) Ion chromatogram of 16 mixed monosaccharide standards; (**B**) ion chromatogram of PKP. The peaks at 2.0 min and 41.0 min correspond to NaOH (solvent) and CH_3_COONa, respectively.

As shown in Figure 3, the sample primarily consists of fructose (27.5 min), glucose (19.5 min), galactose (17.1 min), and arabinose (13.3 min). Ion chromatography analysis revealed that the molar ratio of the polysaccharides was composed of fructose (83.8%), glucose (10.6%), galactose (3.2%), and arabinose (2.4%) (Table 2). No amino sugars or uronic acids were detected. Among these, fructose predominated (218.05 μg/mg), followed by glucose (27.56 μg/mg).

**Figure 4:**
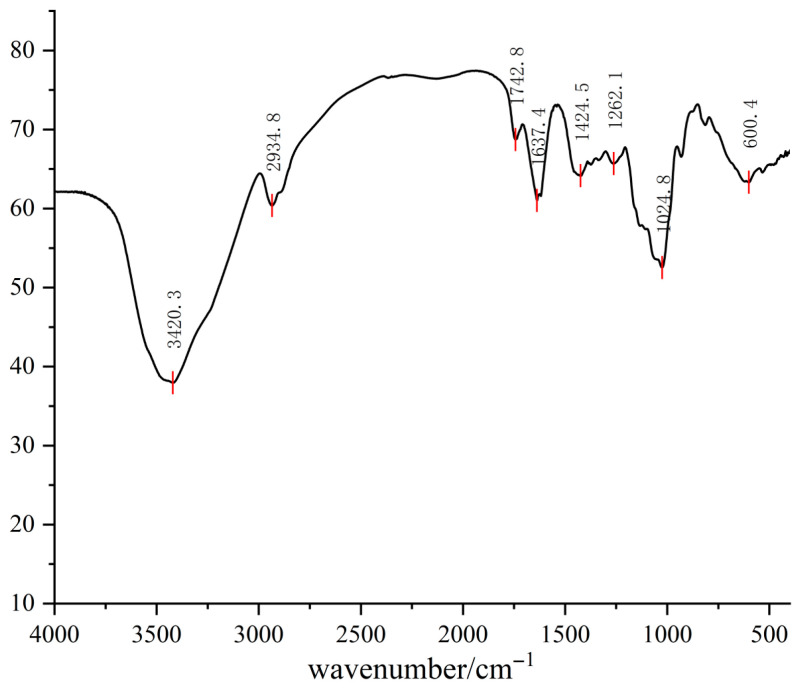
Infrared spectrum of PKP.

The FTIR spectrum (Figure 4) displayed a broad absorption band at 3420.3 cm^−1^ and a peak at 2934.8 cm^−1^. A distinct signal at 1742.8 cm^−1^ was recorded, corresponding to the stretching vibration of carbonyl groups (C=O). The band at 1637.4 cm^−1^ was attributed to cyclic ether groups or bound water. Strong signals in the 1200–1000 cm^−1^ region, particularly at 1024.8 cm^−1^, confirmed the presence of glycosidic linkages.

### 3.2. Characterization of PKS

Systematic characterization of PKS by LC–MS led to the tentative identification of 13 compounds (Table 3). These included several steroidal saponins, together with minor amounts of coumarin- and phospholipid-related compounds. In both positive and negative ion modes, representative steroidal saponins such as Protodioscin (**2**), Pseudoprotodioscin (**5**), and Dioscin (**7**) were detected.

In the retention time range of 14–15 min, compounds such as Pseudoprotodioscin (**5**), Pennogenin-3-O-chacotrioside (**6**), and Prosapogenin A of dioscin(**8**) were detected. The MS/MS spectra showed specific fragment ions at *m*/*z* 415, 253, and 577, indicating the presence of glycosylated saponin structures. Although components **1** and **7** had high-confidence matches (over 96%) in the SIRIUS database, further confirmation was not possible because authentic standards were not available. Component **11**, producing fragment ions at *m*/*z* 415 and 315, could not be definitively identified with the available data.

**Figure 5:**
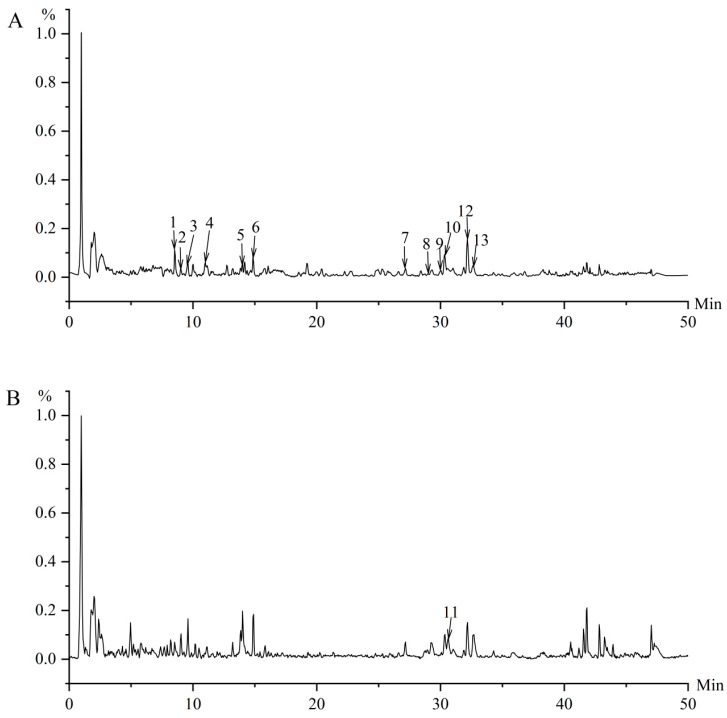
Total ion current chromatograms of PKS. (**A**) Positive mode, (**B**) negative mode. The numbers 1–13 correspond to the identified compounds listed in Table 3.

Additionally, while the absence of some authentic standards prevented the absolute confirmation of all isomers, the identification was supported by high-confidence matches (>96%) in the SIRIUS database for components 1 and 7. Based on the relative peak heights in the TIC chromatogram (Figure 5), Protodioscin (**2**), Pseudoprotodioscin (**5**), and Dioscin (**7**) were identified as the predominant constituents within the PKS fraction. Quantitative analysis further revealed that the concentration of Dioscin (**7**) in the PKS fraction was 0.084% (*w*/*w*). The high signal intensities of these major peaks, combined with the quantified marker, indicate that these specific saponins represent the dominant chemical profile of PKS and are likely the primary contributors to the observed anti-amyloid effects.

### 3.3. Effects of PKP and PKS on Locomotor Behavior in AlCl_3_-Induced Zebrafish

**Figure 6:**
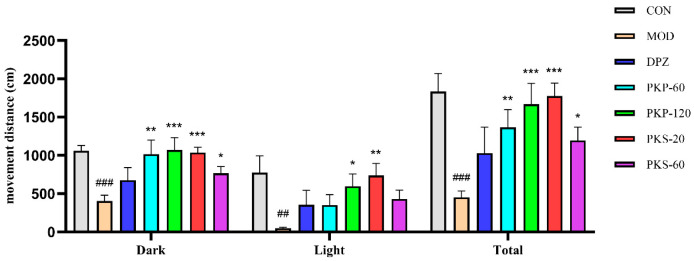
Effect of PKP and PKS on the total distance traveled in zebrafish induced by AlCl_3_ (n = 18; ## *p* < 0.01 and ### *p* < 0.001 compared with the CON group; * *p* < 0.05, ** *p* < 0.01 and *** *p* < 0.001 compared with the MOD group).

The light/dark cycle test was employed to evaluate the locomotor activity of zebrafish subjected to AlCl_3_ (Figure 6). When contrasted with the CON group, zebrafish in the MOD group displayed significant decreases in movement distance across the dark phase, light phase, and overall observation period; specifically, the total distance traveled diminished from 1832.47 cm in the CON group to 443.89 cm in the MOD group. This finding suggests considerable behavioral deficits following exposure to acidic AlCl_3_.

Compared with the MOD group, the DPZ group showed partial improvement, with a total movement distance of 1027.75 cm, although this value remained lower than that of the CON group. Both PKP treatment groups showed significant improvements in locomotor activity. PKP-120, in particular, exhibited the most significant enhancement in total movement distance, reaching 1658.43 cm, thereby surpassing all other groups. The PKS treatment groups also demonstrated improved locomotor behavior, yet the response pattern diverged from that observed with PKP. Specifically, PKS-20 significantly increased the total movement distance, whereas the improvement in the PKS-60 group was less marked. These findings indicate that both PKP and PKS mitigated AlCl_3_-induced locomotor deficits in zebrafish, with PKP yielding a more uniform improvement across all administered doses.

### 3.4. Effects of PKP and PKS on Zebrafish AChE Activity Levels

**Figure 7:**
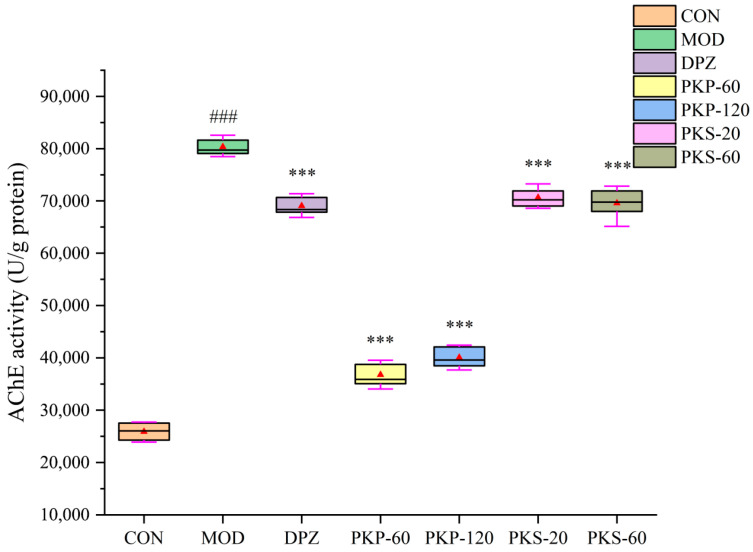
Effect of PKP and PKS on AChE activity in zebrafish induced by AlCl_3_ (n = 3 biological replicates, 30 larvae per replicate; ### *p* < 0.001 vs. CON group; *** *p* < 0.001 vs. MOD group).

As shown in Figure 7, AChE activity within the MOD group exhibited a statistically significant elevation when compared to the CON group. Specifically, the AChE activity in the MOD group attained a value of 80,357.19 U/g protein, which represents an approximate three-fold increase relative to the CON group’s value of 25,893.79 U/g protein (*p* < 0.001). Subsequent to PKP treatment, a notable mitigation in the heightened AChE activity was observed. The PKP-60 and PKP-120 groups displayed AChE activity reductions to 36,764.75 and 40,072.36 U/g protein, respectively (both *p* < 0.001 vs. MOD), with PKP-60 showing the lowest enzyme activity among all treatment groups. By comparison, the PKS-20 and PKS-60 groups also exhibited lower AChE activity, with the magnitude of reduction comparable to that observed in the DPZ group.

### 3.5. Effects of PKP and PKS on Aβ Aggregation in Zebrafish

**Figure 8:**
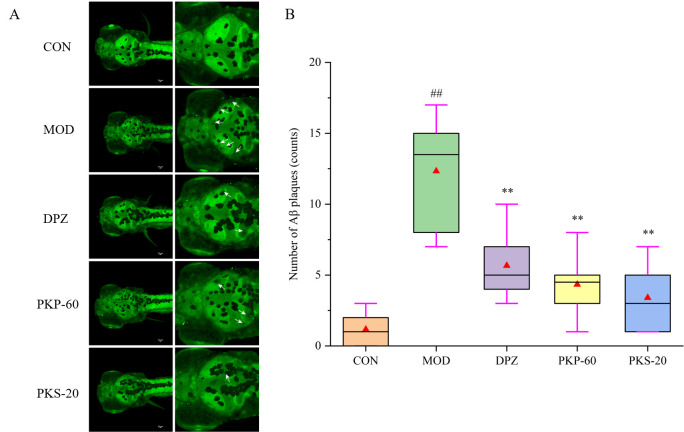
Effect of PKP and PKS on Aβ aggregation and plaque formation in zebrafish induced by AlCl_3_ (*n* = 6). (**A**) Representative fluorescence images of Aβ plaques (indicated by white arrows) in the brains of zebrafish; (**B**) Quantitative analysis of the number of Aβ plaques. ## *p* < 0.01 vs. CON group; ** *p* < 0.01 vs. MOD group. The red triangles represent the mean values.

Thioflavin S staining was used to visualize the distribution of Aβ aggregates in zebrafish brain tissue (Figure 8). The CON group exhibited no discernible fluorescence. In contrast, the AlCl_3_-induced MOD group demonstrated a marked increase in fluorescent puncta (indicated by arrows in Figure 8). Compared with the MOD group, both the PKP-60 and PKS-20 treatments significantly reduced the accumulation of Aβ aggregates (*p* < 0.01). Specifically, PKS-20 exhibited the most pronounced effect, reducing the plaque burden by 62%, while PKP-60 also resulted in a clear reduction in fluorescence intensity.

### 3.6. Effects of PKP and PKS on Zebrafish Brain Cell Apoptosis

**Figure 9:**
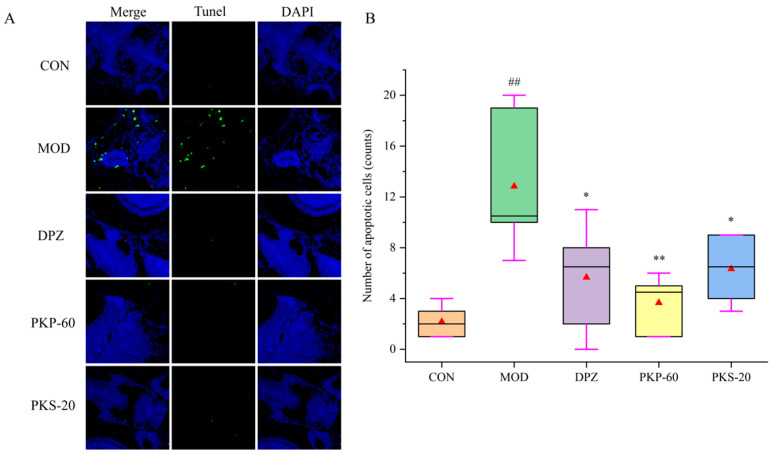
Effect of PKP and PKS on neuronal apoptosis in zebrafish induced by AlCl_3_ (*n* = 6). (**A**) Representative TUNEL staining images in the zebrafish brains (green fluorescence indicates apoptotic cells; blue fluorescence indicates DAPI-stained nuclei), scale bar = 50 μm; (**B**) Quantitative analysis of the number of apoptotic cells. ## *p* < 0.01 vs. CON group; * *p* < 0.05 and ** *p* < 0.01 vs. MOD group. The red triangles represent the mean values.

TUNEL staining was performed to evaluate cell apoptosis in the zebrafish brain (Figure 9). The number of TUNEL-positive cells in the MOD group was significantly higher than that in the CON group (*p* < 0.01). Compared with the MOD group, a reduction in apoptotic cells was observed in the DPZ, PKP-60, and PKS-20 groups. Specifically, PKP-60 exhibited the most pronounced effect, reducing the number of apoptotic cells by 68%, while the anti-apoptotic effect of PKS-20 was relatively less marked.

## 4. Discussion

This research involved the isolation of two principal fractions, polysaccharides (PKPs) and steroidal saponins (PKSs), followed by an assessment of their impacts within an AlCl_3_-induced zebrafish model. Although both fractions improved locomotor deficits and cognitive impairments, their effects exhibited divergence, thereby implying the existence of distinct operational mechanisms.

HPGPC analysis showed that PKP in the present study differed from the high-molecular-weight polysaccharides commonly reported in Polygonatum. Instead, PKP was dominated by a low-molecular-weight fraction at 1890 Da, accounting for 83.82% of the total peak area. This observationmay be associated with to processing-induced depolymerization [27], specifically the repeated steaming and drying cycles, which may lead to the cleavage of glycosidic bonds. Furthermore, variations in the source of raw materials might have also contributed to this specific molecular weight profile. This difference may be related to traditional processing, as previous studies have reported that *Polygonatum* polysaccharides undergo marked structural changes during processing [28]. In the present study, fructose and glucose were the predominant monosaccharides in PKP, accounting for 83.8% and 10.6%, respectively. This composition indicates that PKP mainly consisted of fructose-rich saccharide residues.

The FTIR spectrum of PKP (Figure 4) further confirmed these typical structural features. The broad absorption band in the range of 3600–3200 cm^−1^ (centered at 3420.3 cm^−1^) and the peak at 2934.8 cm^−1^ were assigned to the stretching vibrations of hydroxyl (O–H) and C–H groups, respectively [29,30]. The weak absorption band at 1742.8 cm^−1^ was attributed to the C=O stretching vibration of O-acetyl groups. Notably, the absorption at 1637.4 cm^−1^ was primarily attributed to the bending vibration of bound water. Although this region is sometimes associated with amide groups, our previous UV-Vis analysis (Section 2.2.1) confirmed the absence of protein contamination, supporting the assignment of this peak to water molecules [30]. Furthermore, the strong absorption at 1024.8 cm^−1^ (within the 1200–1000 cm^−1^ range) is characteristic of C–O and C–O–C stretching vibrations, confirming the presence of glycosidic linkages [29,30]. The weak peak at 1262.1 cm^−1^ was associated with the polysaccharide fingerprint region [31]. Overall, the FTIR results were consistent with the structural characteristics of a purified polysaccharide.

Polysaccharides from natural sources are known to exhibit antioxidant and neuroprotective activities. Similar observations have been reported for *Ziziphus jujuba* Mill., in which polysaccharides were considered important contributors to anti-aging activity [32]. Our results further suggest that the low-molecular-weight fraction of PKP may contribute to its bioactivity. On the saponin side, LC-MS analysis of PKS identified 13 distinct steroidal saponins, including Protodioscin. The observed MS/MS fragment ions and these fragmentation patterns align with the known cleavage patterns of steroidal saponins [33], providing a reliable basis for the tentative identification of these compounds despite the lack of authentic standards.

Bioactively, the two fractions behaved quite differently, demonstrating distinct pharmacological tendencies. PKP seemed to focus its efforts on the cholinergic system. In fact, the PKP-60 group showed stronger AChE inhibition than DPZ, the current clinical standard. Donepezil has been reported to induce changes in heart rate and PR interval in clinical settings [34]. Previous studies have suggested that natural polysaccharides may improve cholinergic dysfunction through AChE-related pathways [35]. Under the present experimental conditions, PKP exerted a more pronounced effect in normalizing AChE activity than PKS, with the PKP-60 group even outperforming the positive control (DPZ). Notably, while PKP-120 demonstrated the most significant recovery in locomotor distance, the PKP-60 group showed a slightly more pronounced inhibition of AChE activity. This slight increase in AChE activity at the highest dose (120 μg/mL) compared to the intermediate dose (60 μg/mL) might suggest a plateau effect or a dose-specific feedback regulation within the cholinergic system, which did not impede the overall recovery of locomotor functions. The present results are in agreement with previous studies indicating that natural polysaccharides may alleviate cholinergic dysfunction through AChE-related regulation.

Aβ aggregates are a defining characteristic of AD pathology [36]. In the present study, the marked increase in Thioflavin S-positive fluorescent puncta in the MOD group confirms considerable amyloid plaque formation subsequent to aluminum exposure, which aligns with the observed behavioral deficits. By contrast, PKS showed a stronger effect on amyloid pathology. Thioflavin S staining showed that the PKS-20 group reduced Aβ aggregate accumulation by 62%, indicating a clearer effect on amyloid-related changes. Interestingly, the efficacy of PKS was more pronounced at 20 μg/mL than at 60 μg/mL, which might be attributed to potential sedative or mild toxic effects of high-concentration steroidal saponins in zebrafish, partially masking their neuroprotective benefits. Previous studies have reported that saponin-like compounds may reduce Aβ production through pathways associated with PPARγ activation [37]. The anti-amyloid effect of PKS observed in the present study may therefore be related to the biological activity of its steroidal saponins, although the exact mechanism remains unclear.

Neuronal apoptosis is a pathological hallmark of AlCl_3_-induced neurotoxicity. In our study, the significantly higher number of TUNEL-positive cells in the MOD group indicates marked neuronal damage following aluminum exposure. In the TUNEL assay, PKP-60 reduced apoptotic cells by 68%, indicating a stronger anti-apoptotic effect than that observed in the low-dose saponin group and the positive control. These findings are consistent with observations of other natural bioactive compounds. Earlier studies have shown that polysaccharides from related *Polygonatum* species can attenuate neuronal apoptosis through survival-related pathways such as PI3K/Akt [38]. Since no pathway-specific analysis was performed here, this can only be considered indirect support. Nevertheless, the present results indicate that PKP contributed more strongly to neuronal survival than PKS in this model.

Overall, the present results suggest that *Polygonatum kingianum* is a potential source of multi-component neuroprotective ingredients. Related bioactivities have also been reported for natural products such as *Panax notoginseng*, licorice, and astragalus [39,40,41]. In the present study, the two major fractions of PK showed different functional tendencies: PKP was more closely associated with cholinergic regulation and the inhibition of neuronal apoptosis, whereas PKS showed a stronger effect on Aβ aggregation.

## 5. Limitations and Future Direction

This study compared the individual neuroprotective effects of PKP and PKS, but their potential synergistic effects were not evaluated. In addition, the current work mainly focused on behavioral and pathological changes, without further investigation of the underlying molecular mechanisms. Future studies should therefore examine the combined effects of PKP and PKS and perform more detailed mechanistic analyses, such as Western blot or RT-qPCR, to clarify the pathways associated with cholinergic regulation, amyloid pathology, oxidative stress, and apoptosis.

## 6. Conclusions

In conclusion, both the polysaccharide (PKP) and saponin (PKS) fractions from *Polygonatum kingianum* showed neuroprotective effects in the AlCl_3_-induced zebrafish model. Our results reveal a clear divergence in their mechanisms of action: PKP primarily alleviates cognitive deficits by regulating the cholinergic system and inhibiting neuronal apoptosis, whereas PKS exhibits superior efficacy in mitigating Aβ aggregation. These findings indicate that the two major fractions of *Polygonatum kingianum* differ in their neuroprotective characteristics and may alleviate AD-like pathological changes through different functional tendencies. This study provides experimental support for the potential use of *Polygonatum kingianum* as a source of food-derived bioactive ingredients. Furthermore, it establishes a scientific basis for future investigations into the potential synergistic effects of combined PKP and PKS fractions, which may offer more comprehensive multi-target protection against neurodegeneration.

## Figures and Tables

**Table 1:** Molecular weight determination results.

Rt (min)	Mp (Da)	Mw (Da)	Mn (Da)
33.530	41,321	41,254	40,021
39.983	1890	1930	1847

Mn represents the number-average molecular weight; Mp represents the peak molecular weight; Mw represents the weight-average molecular weight. The signal at 42.8 min corresponds to the system peak.

**Table 2:** Composition of monosaccharides in PKP.

Name	Abbreviation	Peak Area	Retention Time (min)	Molar Ratio	Content (μg/mg)
Fucose	Fuc	0	5.909	0.000	0.000
Amino-galactose hydrochloride	GalN	0	11.542	0.000	0.000
Rhamnose	Rha	0	12.125	0.000	0.000
Arabinose	Ara	1.756	13.259	0.024	5.279
Glucosamine hydrochloride	GlcN	0	14.684	0.000	0.000
Galactose	Gal	1.793	17.15	0.032	8.371
Glucose	Glc	9.689	19.517	0.106	27.557
N-Acetyl-D-glucosamine	GlcNAc	0	20.959	0.000	0.000
Xylose	Xyl	0	22.925	0.000	0.000
Mannose	Man	0	23.867	0.000	0.000
Fructose	Fru	27.517	27.45	0.838	218.054
Ribose	Rib	0	28.50	0.000	0.000
Galacturonic acid	GalA	0	43.125	0.000	0.000
Guluronic acid	GulA	0	43.759	0.000	0.000
Glucuronic acid	GlcA	0	45.584	0.000	0.000
Mannuronic acid	ManA	0	47.792	0.000	0.000

**Table 3:** Qualitative results of PKS.

No.	Rt (min)	Formula	Theoretical (*m*/*z*)	ESI+			ESI−			MS/MS Fragments (*m*/*z*)	Identification	CAS
				Observed *m*/*z*	Mass Error (ppm)	Score	Observed *m*/*z*	Mass Error (ppm)	Score			
1	8.520	C_14_H_29_NO_4_	275.210	276.2179 ([M+H]^+^)	3.31	96.66	274.2029 ([M-H]^−^)	1.79	98.84	(+) 149, 117, 102 (−) 274, 200, 126	-	-
2	8.987	C_51_H_84_O_22_	1048.545	1031.5423 ([M+H-H_2_O]^+^)	0.54	73.83	1047.5373 ([M-H]^−^)/1093.5428 ([M+FA-H]^−^)	−0.54	91.00	(+) 869, 725, 578, 415, 379, 253, 129 (−) 1047, 901, 755, 593, 558, 404, 101	Protodioscin	55056-80-9
3	9.597	C_45_H_74_O_18_	902.488	885.4843 ([M+H-H_2_O]^+^)	−0.03		901.4794 ([M-H]^−^)/947.4849 ([M+FA-H]^−^)	−1.14	98.68 91.47	(+) 723, 579, 397, 253, 197, 157, 129 (−) 901, 755, 575, 352, 143	Diosgenin saponin C	142759-74-8 /185432-00-2
4	11.142	C_16_H_16_O_6_	304.095	305.1027 ([M+H]^+^)	2.14	99.50 79.85	-	-	-	(+) 245, 175, 128, 107	Phenolic acid derivative	152615-15-1 /152615-14-0
5	14.005	C_51_H_82_O_21_	1030.535	1031.5422 ([M+H]^+^)/1048.5690 ([M+NH4]^+^)	0.12	98.92	1029.5261 ([M-H]^−^)/1075.5325 ([M+FA-H]^−^	−0.82	81.79/80.09	(+) 1031, 869, 723, 577, 415, 293, 129 (−) 1029, 883, 737, 671, 575, 433, 342, 205, 143	Pseudoprotodioscin	102115-79-7
6	14.848	C_45_H_72_O_17_	884.477	885.4848 ([M+H]^+^)	0.79	99.01	883.4688 ([M-H]^−^)	−1.01	86.33	(+) 723, 577, 415, 253, 129 (−) 883, 737, 557, 372, 247, 205, 179	Pennogenin-3-O-chacotrioside	55916-52-4
7a	27.157 *	C_42_H_70_O_13_	782.482	-	-	-	781.4738 ([M-H]-)/827.4793 ([M+FA-H]^−^)	−1.1	96.97	(−) 781, 619, 457, 379, 221, 161, 101	Proposed saponin derivative	-
7b	27.157 *	C_45_H_72_O_16_	868.482	869.4898 ([M+H]^+^)	-	-	913.4798 ([M+FA-H]^−^)	−0.7	78.79	(−) 867, 721, 575, 509, 415, 205, 163	Dioscin *	19057-60-4
8	28.983	C_39_H_62_O_12_	722.424	723.4313 ([M+H]^+^)	−0.73	87.41	767.4215 ([M+FA-H]^−^)	−1.18	88.82	(−) 767, 721, 575, 518, 414, 343, 205, 134	Prosapogenin A of dioscin	19057-67-1
9	30.014	C_21_H_22_O_7_	386.137	404.1709 ([M+NH4]^+^)	0.16	81.31	-	-	-	(+) 404, 327, 283, 227, 175	Coumarin derivative	73069-27-9
10	30.342	C_26_H_50_NO_7_P	519.333	520.3409 ([M+H]^+^)	1.93	97.92	564.3305 ([M+FA-H]^−^)	−0.49	98.36	(+) 337, 258, 184, 124, 104 (−) 504, 279, 224, 168, 152	2-linoleoyl-sn-glycero-3-phosphocholine	-
11	30.636	C_30_H_40_N_6_O_7_	596.296	579.2937 ([M+H-H_2_O]^+^)	-	-	595.2892 ([M-H]^−^)	0.99	98.51	(+) 561, 425, 337, 263, 155, 109 (−) 595, 415, 315, 279, 241, 152	-	-
12	32.170	C_24_H_50_NO_7_P	495.333	496.3416 ([M+H]^+^)	3.29	93.46	540.3308 ([M+FA-H]^−^)	−0.41	98.98	(+) 496, 313, 184, 124, 104 (−) 540, 480, 255, 224, 168, 152	1-Palmitoyl-Lysolecithin	14863-27-5
13	32.592	C_25_H_49_O_12_P	572.296	555.2936 ([M+H-H_2_O]^+^)	0.95	91.78	571.2886 ([M-H]^−^)	−0.64	94.02	(+) 537, 393, 313, 239, 155, 109 (−) 571, 391, 315, 255, 223, 152	1-palmitoyl-sn-glycero-3-phosphoinositol	-

Note: *m*/*z* values in parentheses (e.g., [M-H]^−^, [M+FA-H]^−^, [M+NH_4_]^+^) represent the observed adduct ions in ESI+ or ESI− modes, which account for the multiple mass values reported for a single compound. * Compound **7b** (Dioscin) was identified and quantified using an authentic standard; its content in PKS was determined to be 0.084% (*w*/*w*). Compounds were identified by matching experimental HRMS and MS/MS spectra with the Agilent PCDL library and SIRIUS 5.0 and were further cross-referenced with the known chemical constituents of the genus Polygonatum reported in previous studies to ensure chemotaxonomic consistency. The symbol “-” indicates that no direct library match was found; in these cases, molecular formulas (e.g., Peak 4) were proposed based on high-resolution mass data with a mass error < 5 ppm. Identifications for co-eluting isomers (e.g., Peak 7a and 7b) are tentative where authentic standards were unavailable.

## Data Availability

The data presented in this study are available on request from the corresponding author. The data are not publicly available at this time as they also form part of an ongoing broader study.
